# Monocytes from Depressed Patients Display an Altered Pattern of Response to Endotoxin Challenge

**DOI:** 10.1371/journal.pone.0052585

**Published:** 2013-01-03

**Authors:** Lucia Lisi, Giovanni Camardese, Mariangela Treglia, Giuseppe Tringali, Cinzia Carrozza, Luigi Janiri, Cinzia Dello Russo, Pierluigi Navarra

**Affiliations:** 1 Institute of Pharmacology, Catholic University Medical School, Rome, Italy; 2 Institute of Psychiatry and Psychology, Catholic University Medical School, Rome, Italy; 3 Institute of Biochemistry and Clinical Biochemistry, Catholic University Medical School, Rome, Italy; University of Leicester, United Kingdom

## Abstract

It is now well established that major depression is accompanied and characterized by altered responses of the immune-inflammatory system. In this study we investigated the pro-inflammatory activation of monocytes isolated from depressed patients as a parameter not influenced by such confounds as the time of day, the nutritional and exercise status or the age and gender of patients. Monocytes from depressed patients and from healthy controls were isolated in vitro; after 24-h incubation under basal conditions, cells were exposed for 24-h to 100 ng/ml of endotoxin (bacterial lipopolysaccharide, LPS). We found that monocytes from drug-free depressed patients and controls release the same amounts of prostaglandin E2 (PGE2) under basal conditions, whereas monocytes from patients are dramatically less reactive to LPS (8.62-fold increase vs previous 24 hrs) compared to healthy controls (123.3-fold increase vs previous 24 hrs). Such blunted prostanoid production was paralleled by a reduction in COX-2 gene expression, whereas other pro-inflammatory mediators, namely interleukin-1β (IL-1 β) and -6 (IL-6) showed a trend to increased gene expression. The above changes were not associated to increased levels of circulating glucocorticoids. After 8 months of antidepressive drug treatment, the increase in PGE2 production after the endotoxin challenge was partially restored, whereas the increase in IL-1 β and -6 levels observed at baseline was completely abolished. In conclusion, our findings show that the reactivity of monocytes from depressed patients might be considered as a marker of the immune-inflammatory disorders associated to depression, although the lack of paired healthy controls at follow-up does not allow to conclude that monocyte reactivity to endotoxin is also a marker of treatment outcome.

## Introduction

Major depressive disorder (MDD) is a severe and potentially debilitating psychiatric illness characterized by significant changes in mood. MDD frequently presents with a chronic course and can result in marked individual suffering and disability [1]. Evidence collected over the last 40 years indicates that disturbances in serotonergic and noradrenergic neurotransmission are a pivotal factor in the pathogenesis of MDD. Although the roles of serotonin and noradrenalin neuronal systems have extensively been studied, certain aspects of MDD patho-physiological mechanism remain to be clarified [2].

Proinflammatory cytokines are involved in a variety of behavioral, neuroendrocrine and neurochemical alterations that accompany MDD [3]. Accordingly, elevations in circulating pro-inflammatory cytokines and other inflammation-related proteins are a well-documented finding [4]. In particular, prostaglandin (PG) E2 is a product of the arachidonic acid cascade that enhances its own production but suppresses acute inflammatory mediators, resulting in its predominance at late/chronic stages of immune response [5]. Increased PGE2 levels were observed in saliva [6], cerebrospinal fluid and serum [7], [8] of depressed patients.

So far, no standardized test has been developed to use plasma, serum or saliva levels of inflammatory mediators as a possible marker of illness or treatment response in MDD, probably because of methodological difficulties in the measurement of these parameters. In fact, cytokines and PGE2 concentrations in biological fluids are influenced by a huge variety of factors, including timing of measurement, nutritional and exercise status [9].

The activation of hypothalamus-pituitary-adrenal (HPA) axis is a most extensively investigated parameter in MDD; an altered function of the HPA axis as well as the corticotrophin releasing hormone (CRH) system have been consistently found in subjects with MDD [10]. Patients with MDD have elevated cortisol levels in plasma as well as elevated cortisol response to combined test dexamethasone/CRH [11]. However, the activation of HPA axis in depressed patients appears to be dependent on several factors, including melancholic and atypical features, chronicity, cytokine involvement and genetic traits; therefore, depressed patients do not show an HPA axis hyperactivity in as much as 27–32% of the cases [12], [13].

In this study, we investigated the proinflammatory activation of monocytes isolated from unmedicated depressed patients and in healthy controls. We also evaluated the status of activation of HPA axis by salivary cortisol circadian rhythm and after low dose dexamethasone suppression test.

## Materials and Methods

### Subjects

Ten drug-free outpatients who met the DSM-IV-TR criteria for MDD were recruited at the Day Clinic of the Institute of Psychiatry of the Catholic University in Rome during a Major Depressive Episode (MDE), without psychotic features. The Mini-International Neuropsychiatric Interview (M.I.N.I.) was conducted to confirm the diagnosis of MDD and rule out bipolarity or other exclusionary comorbid axis I disorders [14]. Patients underwent psychiatric, physical and laboratory examinations to evaluate blood cell count as well as liver, kidney and thyroid function. Exclusion criteria were the presence of: concurrent psychiatric illnesses (psychotic disorders, eating disorders, personality disorders, mental retardation, anxiety disorders -except for specific phobia-, mental disorders due to a general medical condition); smoking history or substance abuse in the previous six months; history of seizures; current liver, kidney or heart disease; neoplastic diseases; neurological disorders; chronic pain; allergic reactions or infection in the previous four weeks; AIDS; autoimmune diseases. Underweight (BMI<18.5) or overweight (BMI≥25) patients suffering from endocrine, immune or metabolic disorders were also excluded. Similarly, individuals who attempted suicide in the previous six months were excluded. However, it was not possible to rule out, as possible causes of immune system alterations, the effects exerted by sleep deprivation and physical or psychological stress, which are all frequent in depressed patients. In the patient group, 6 were drug-naïve and 4 had taken antidepressants that had been discontinued at least 2 weeks prior to enrolment.

All patients underwent a psychometric assessment by the same trained physician, blind to diagnosis, using the Italian version of the 21-item Hamilton Depression Rating Scale (HDRS). This scale provides a simple and reliable way to quantitatively assess the magnitude and frequency of the patient's depressive symptoms and to document changes in time. Of the 21 items, 10 are rated on a 5-point scale (0–4), 2 on a 4-point scale (0–3) and the remaining 9 on a 3-point scale (0–2). Levels of severity for most items are well defined. The following cut-off scores identify growing levels of depression severity: 8–17 mild depression; 18–24 moderate depression; ≥25 severe depression [15].

Before starting the pharmacological treatment a blood sample for laboratory testing was collected. Blood sampling was carried out at 8 a.m., after an overnight fasting. All patients were then treated with an antidepressant mono-therapy. Escitalopram, a Selective Serotonin Reuptake Inhibitor (SSRI), was started at the dose of 10 mg per day. Seven out of 10 patients required a dose adjustments to 20 mg per day. Both treatment schedules are included in the SmPC of the drug. Every patient was regularly reviewed and HDRS scale was administered by the same rater, blind to treatment, at each follow-up visit (after two weeks, one month, three months and eight months) to assess treatment outcome. We considered ‘responders’ all subjects with a 50% decrease on total HDRS score and the dosage of escitalopram was increased up to 20 mg per day, during the first two weeks, if a treatment response was not achieved. During the last visit (after eight months), a second blood sample for laboratory testing was collected as previously described.

Finally, 10 subjects were recruited as healthy controls with a BMI between 18.5 and 25 kg/m^2^ and without any kind of medical condition. Based on the M.I.N.I., axis I psychiatric disorders were excluded. They were non-smokers neither receiving medication nor abusing of other substances.

The study was approved by the institutional review board. All subjects gave their informed consent and possible side effects were fully explained.

### Isolation of peripheral blood mononuclear cell subsets

Human peripheral blood mononuclear cells (PBMC) were obtained from EDTA peripheral blood by density gradient centrifugation using the Ficoll-Paque Premium. Blood cell count from both the depressed patients and the controls did not show abnormal values and no significant differences were observed between groups for monocyte count (0.36±0.09 vs. 0.46±0.15×10^6^ cells/ml; F = 3.54, n.s. difference). After two washes with balanced salt solution, the cells were resuspended in culture medium RPMI-1640 supplemented with 2 mM L-glutamine, 100 UI/ml penicillin/streptomycin and 10% heat inactivated FCS (Gibco Brl, Invitrogen Corporation, Paisley, UK) and were plated at density of 4×10^6^ cells/well on a highly immunoadsorbent plates to facilitate monocyte adhesion (Corning CellBIND Surface). After 3 hours of incubation at 37°C in a 95% O2/5% CO2 humidified atmosphere, lymphocytes were removed, by washing the plates twice with PBS and monocytes were further incubated overnight. After this incubation period, monocytes were treated with medium alone RPMI-1640 or with 100 ng/ml endotoxin bacteria (LPS) for 24 h; the parameters concentration and time of exposure were set according to our previous experience with other cellular models, as well as to data from literature [16]. At the end of the experiment the medium was collected for the PGE2 assessment.

### mRNA analysis in real time PCR

Total cytoplasmic RNA was extracted using the RNeasy Micro kit (Qiagen, Hilden, Germany), which included 15 min DNAse treatment. RNA concentration was measured using the Quant-iT™ RiboGreen® RNA Assay Kit (Invitrogen Corporation, Carlsbad, CA, USA). A standard curve in the range of 0–10 ng was run in each assay using 16S and 23S ribosomal RNA (rRNA) from E. Coli as standard and provided by the kit. Aliquots (0.1 µg) of RNA were converted to cDNA using random hexamer primers. Quantitative changes in mRNA levels were estimated by real time PCR (Q-PCR) using the following cycling conditions: 40 cycles of denaturation at 95°C for 20 s; annealing at 60°C for 30 s; and extension at 72°C for 30 s; using the Brilliant SYBR Green QPCR Master Mix 2× (Stratagene, La Jolla, CA, USA). PCR reactions were carried out in a 20 µL reaction volume in a MX3000P real time PCR machine (Stratagene). Primers used for the evaluation of gene expression are b-actin 471F (5′-ACG TTG CTA TCC AGG CTG TCC AT-3′) and 711R (5′-TTA ATG TCA CGC ACG ATT TCC CGC-3′) which yield a 241 base pair (bp) product; human COX2 1381F (5′-TTG CTG GCA GGG TTG CTG GTG GTA-3′), and 1466R (5′CAT CTG CCT GCT CTG GTC AAT CGA A-3′) which yield a 86 bp product; human IL-6 49F (5′GGC TCA TTC TGC CCT CGA GCC-3′) and 148R (3′GGA CCG AAG GCG CTT GTG GAG-5′) which yield a 100 bp product; human IL-1β 84F (5′AGC CAT GGC AGA AGT ACC GT3′) and 302R (5′TCC ATG GCC ACA ACA ACT GA 3′) which yield a 219 bp product; and human toll-like receptor 4 (TLR4) 25F (5′-GCC ACT GGT CTG CAG GCG TT-3′), and 401R (3′- TAG GAA CCA CCT CCA CGC AGG G-5′) which yield a 377 bp product; Relative mRNA concentrations were calculated from the take-off point of reactions (threshold cycle, Ct) using the comparative quantization method performed by Stratagene software and based upon the −ΔΔCt method. This analysis approximates a given sample's target mRNA (*e.g.* COX2) level relative to the mean of the target mRNA levels in untreated controls (“calibrator” value), thus permitting statistical analysis of deviation from the mean even among the controls. Ct values for b-actin expression served as a normalizing signal. In each assay, the PCR efficiency was also calculated using serial dilution of one experimental sample; efficiency values between 94 and 98% were found for each primer set and taken into account for the comparative quantization analysis [17].

### PGE_2_ assay

PGE_2_ levels in the incubation medium were detected using a specific Enzyme Immunoassay kit (EIA) for PGE_2_, carried out according to the manufacturer's instructions (Assay Design, Inc., Ann Arbor, MI, USA). Briefly, the medium was collected from the monocyte cultures, aliquoted and stored at −80°C until the day of the assay (avoiding repeated freeze-thaw cycles). A standard curve was generated during each assay in the concentration range 7.81–1.000 pg/ml using the PGE_2_ standard provided in the kit and a best fit curve was plotted. The minimum detectable concentration of PGE_2_ was 7.81 pg/ml.

### Salivary cortisol circadian rhythm and after low dose dexamethasone suppression test

Salivary cortisol circadian rhythm and after low dose dexamethasone suppression test was performed in healthy subjects and in MDD patients during the first evaluation and after a 8 months treatment. Salivary samples were collected at 8 a.m., 4 p.m., 11 p.m. using the a commercially available device (Salivette, Sarstedt; cotton swab without preparation). Low dose dexamethasone suppression test was performed administering dexamethasone 1 mg per os at 11 p.m. and collecting salivary samples the following day at 8 a.m. Salivary samples were centrifuged at 4° C for 10 minutes and stored at −20° C until assayed. Salivary cortisol concentration was determined by an elettrochemiluminescence (ECLIA) Roche method on Modular E without pretreatment of the samples.

### Statistical analysis

Results are shown as mean ± standard error of 10 replicates per each group, as indicated in Figure legend. Data were analyzed by ANOVA, Student's t-test or Mann-Whithney U-test where appropriate, using a PrismTM computer program (GraphPad, San Diego, CA, USA). P values<0.05 were considered significant. Stata2 program was used to calculate the study power.

## Results

No significant differences were identified between patients and healthy controls with regard to basic demographic data including age (controls tended to be younger; t test: p = 0.07) and gender. Table 1 shows demographic parameters, psycho-pathological severity and treatment outcome of depressed patients. Most of them showed a mild to moderate depressive symptomatology at basal evaluation. One month after the antidepressant treatment a significant reduction in HDRS scores was observed (data not shown) and 8 patients showed a 50% decrease in their psycho-pathological burden. All of the patients who achieved a response after 1 month of treatment also maintained a favourable clinical outcome during the entire study duration.

**Table 1:** Demographic parameters, psycho-pathological severity and treatment outcome of depressed patients.

*Demographic data*		
	*Patients*	*Healthy controls*
	Mean ± SD(n)	Mean ± SD(n)
Age (years)	48.40±11.13 (10)	39.30±10.06 (10)
Sex (F/M)	8/2	5/5
BMI (kg/m^2^)	24.76±5.59 (10)	23.09±2.03 (10)

*Psychometric parameters*		
HDRS Scale	Mean ± SD(n)	
Basal	18.50±3.41 (10)	<8 (10)
After 8 months	8.50±6.20 (10)	

Data are expressed as the means ± SD (n = 10).

**Figure 1:**
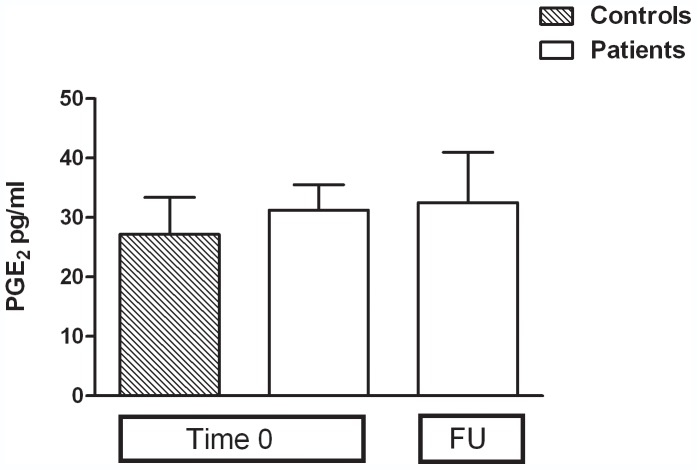
Prostaglandin E_2_ release under basal conditions. Data are expressed as pg/ml, the means ± SEM of 10 subjects per group. FU = Follow up.

**Figure 2:**
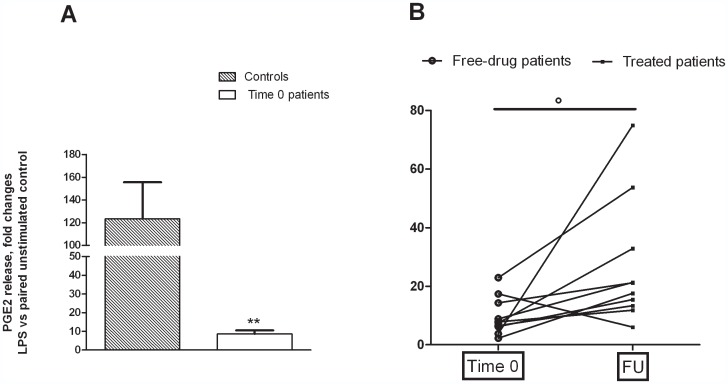
Monocytes were activated for 24 hours with 100 ng/ml endotoxin. Data are expressed as PGE_2_ release, fold changes vs each paired unstimulated control. (**A**) Data are the means ± SEM (n = 10).^**^P<0.01 vs. healthy controls; (**B**) Individual data of patients **°**P<0.05 *vs* patients at time 0; Mann Whitney test. FU = Follow up.

**Figure 3:**
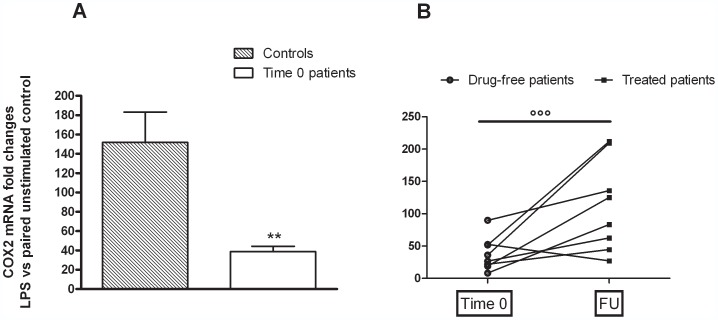
Total cytosolic RNA was prepared used for Q-PCR analysis of the expression of COX2. Data are expressed as fold changes vs each paired unstimulated control, taken as calibrator for comparative quantitation analysis of mRNA levels. Each sample was measured in triplicate. (**A**) Data are the means ± SEM (n = 10).^**^P<0.01 vs. healthy controls; (**B**) Individual data of patients **°°°**P<0.001 vs patients at time 0; Student's t-test. FU = Follow up.

Primary cultures of monocytes from both controls and MDD patients released sizable amounts of PGE2 under basal conditions (Fig. 1). No significant difference was observed either between controls and patients at time 0, or between patients assessed at time 0 and after 8 months of follow-up (Fig. 1). Interestingly, a 24-h incubation with endotoxin caused an average 123.3-fold increase in PGE2 production by monocytes from healthy controls compared to the previous 24 hrs of incubation, whereas only an average 8.62-fold increase was detected in cells harvested from drug-free patients (Controls *vs* patients 0; Student's t-test p = 0.0023; t = 3.546) (Fig. 2). After 8 months of follow-up, the ability of monocytes from MDD patients to respond to the endotoxin challenge appeared to be partially restored (Patients time 0 *vs* patients F.U.; Mann Whitney test, p = 0.0185), since an average 28-fold increase was measured, although such response remained significantly lower compared to healthy controls (Controls *vs* patients F.U.; Student's t-test p = 0.0097; t = 2.895) (Fig. 2). However, no significant correlation was found between the changes in HDRS scores measured in the time-frame 0–8 months and the increased response to endotoxin observed in the same time interval. Similar to PGE_2_, a huge (151.9-fold) increase was observed in COX_2_ gene expression by monocytes from controls, compared to a 38.8-fold increase in monocytes from drug-free patients (Controls *vs* patients 0; Student's t-test p = 0.0024; t = 3.206). This value increased to 98.9-fold at 8-month FU (Fig. 3) (Patients time 0 *vs* patients F.U. Student's t-test p = 0.0005; t = 3.733).

**Figure 4:**
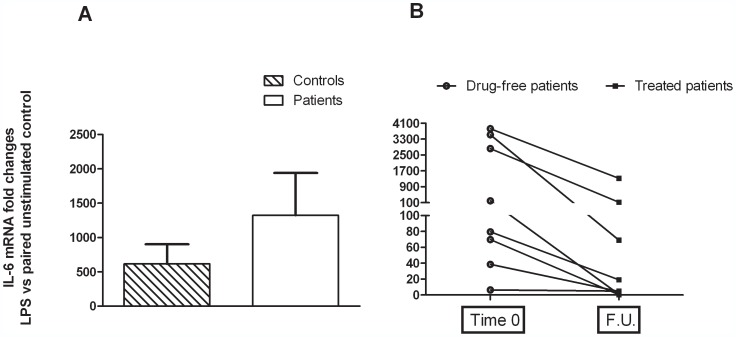
Total cytosolic RNA was prepared used for Q-PCR analysis of the expression of IL-6. Data are expressed as fold changes vs each paired unstimulated control, taken as calibrator for comparative quantitation analysis of mRNA levels. Each sample was measured in triplicate. (**A**) Data are the means ± SEM (n = 10). (**B**) Individual data of patients. FU = Follow up.

**Figure 5:**
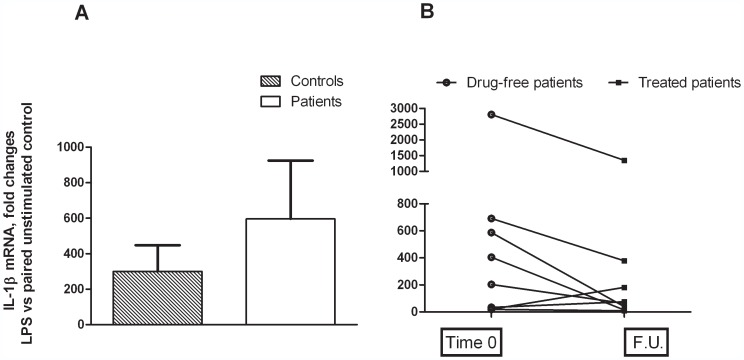
Total cytosolic RNA was prepared used for Q-PCR analysis of the expression of IL-1β. Data are expressed as fold changes vs each paired unstimulated control, taken as calibrator for comparative quantitation analysis of mRNA levels. Each sample was measured in triplicate. (**A**) Data are the means ± SEM (n = 10). (**B**) Individual data of patients. FU = Follow up.

The other mediators of inflammation investigated here, IL-1 β and -6 showed an opposite behaviour compared to the prostanoid system after the LPS challenge. At baseline, cytokine gene expression was markedly increased in drug-naïve MDD patients compared to healthy controls; however, because of the much higher variability of this parameter compared to both PGE2 and COX-2, the difference did not reach statistical significance (Fig. 4 and 5). Likewise, the high variability prevented to show a significant difference between IL-1 β and -6 mRNA levels assessed in patients at time 0 and after FU, although a strong trend to a reduction was observed (Fig. 4 and 5). Limited to the comparison between MMD patients and healthy controls at baseline, we also measured plasma levels of C-Reactive Protein (CRP), and found no significant differences (data expressed as mg/l of human serum CRP, the means ± SEM of 10 observations per group. MMD patients: 1.34±0.30; healthy controls: 1.27±0.59).

In order to investigate the mechanisms underlying the LPS effects in this paradigm, we assessed on our samples the gene expression of TLR-4, i.e. the LPS receptor subtype expressed in human monocytes [18]. We found a tiny increase in gene expression both in Controls and MDD patients, with no significant difference whatsoever (data not shown).

**Figure 6:**
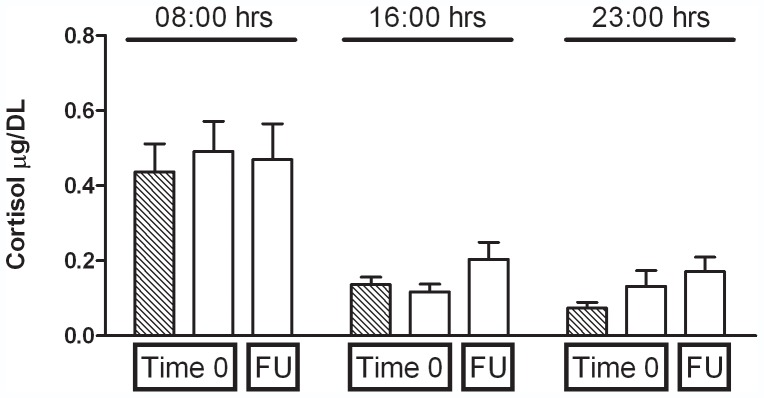
Salivary cortisol levels at 08.00, 16.00 and 23.00 for each experimental group. Data are expressed as µg/Dl, the means ± SEM of 10 subjects per group. FU = Follow up.

It was also interesting to investigate whether the above described changes in inflammatory parameters could be related to changes in HPA axis function in MDD patients; in particular, a putative increase in glucocorticoid levels would tonically inhibit COX-2 gene expression in monocytes. However, we found no differences among the profiles of daily cortisol secretions in healthy controls and MDD patients at time 0, as well as in patients after 8 months of follow-up (Fig. 6). A dexamethasone suppression test carried out in both experimental groups showed that all of the healthy volunteers had the HPA suppressed (11 p.m. salivary cortisol <0.1 µg/dl; salivary cortisol after low dose dexamethasone suppression test <0.06 µg/dl), whereas 3 out of 10 patients resulted to be non-suppressor (11 p.m. salivary cortisol >0.1 µg/dl; salivary cortisol after low dose dexamethasone suppression test >0.06 µg/dl), so they remained after 8 months of follow-up (data not shown).

## Discussion

In this study we were seeking for a marker of inflammation in depressed patients that was possibly not influenced by such confounds as time of day, nutritional and exercise status or the age and gender of patients. With this in mind, we investigated the proinflammatory activation of monocytes isolated by depressed patients. After 24-h incubation under basal conditions, cells were exposed for 24-h to endotoxin. Looking at COX-2 gene expression and prostanoid production, we found that cells from depressed patients are significantly less reactive compared to monocytes obtained from healthy controls. Such huge difference was only partially reversed after 8 months of sustained treatment response. On the contrary, IL-1β and -6 gene expression tended to be higher in patients compared to controls at baseline, and it was blunted after 8 months of treatment with escitalopram, although none of the above changes was statistically different with respect to the controls because of apparently higher variability of cytokine mRNA levels in both experimental groups. We also evaluated the status of activation of HPA axis by salivary cortisol circadian rhythm and after low dose dexamethasone suppression test. In fact, several observations of hypersecretion of CRH and cortisol and of reduced HPA axis feedback from glucocorticoids in MDD patients have been described [19].

The patients described in this report were the first consecutive MDD patients enrolled in a larger clinical trial aimed to investigate putative relationships between disease state and response to treatment on the one hand, and immune-inflammatory parameters on the other hand. This clinical trial is currently ongoing; however, by analysing the first set of data and comparing them with those from matched controls, we observed such a huge difference in the PGE2 response to endotoxin challenge that, despite the preliminary nature of our findings, we though biologically and clinically plausible to draw some conclusions based on such small subset of patients. It was also a statistically plausible approach: with a population of 20 subjects, the study has a power higher than 95% to detect a difference between the two study groups, as far as PGE2 production and COX2 gene expression are concerned.

From the viewpoint of HPA axis function, our MDD patients also appear to be representative of a larger population. In fact, 3 (30%) out of 10 patients not suppressed at the dexamethasone suppression test; this percentage is consistent with most of the data reported in the literature. Vasavada and collaborators showed that non suppression occurred in 27% of patients with MDD and in 43% of bipolar patients during depression phase [13]; similar data have been reported by the group of Iacovides [12].

The profile of IL-1β and -6 gene expression in this experimental paradigm was diverging from those of COX-2 and PGE2, since the cytokine tended to be expressed to a higher level in MDD patients at baseline compared to controls, and it was blunted rather than increased by SSRI treatment. These findings are in keeping with a large evidence reported in the literature [20], [21], 22. However, the very high variability in cytokine mRNA levels prevented to achieve statistically significant differences among experimental groups. As mentioned before, this study is still ongoing at present; a larger sample of population will probably allow us to draw a conclusion about the putative different regulation of IL-6 gene expression in MDD patients.

The pattern of inflammatory mediator gene expression and production in blood cells from MDD patients has been recently investigated by Myint and collaborator [23]. These authors showed that a 72-hr exposure to 5 µg/ml endotoxin induced significantly higher production of both IFN-γ and IL-10 in blood cells from normal controls compared to depressed patients. Based on these findings, these authors postulate that depressed patients present an increased immune-inflammatory response under basal conditions, and the reduced response of blood cells to *in vitro* stimulation reflects an exhaustion of cell function subsequent to sustained hyperactivity. This hypothesis implies that a difference in the production of pro-inflammatory mediators should be already present under basal conditions; however, we observed no difference in baseline PG production between MDD patients and healthy controls. The lack of differences we observed under basal conditions seems to contradict the hypothesis by Myint and colleagues, but it might also be explained by the 24-hr washout we carried out before starting the experiments.

The findings by Myint and colleagues, along with those presented here, for the first time show a different regulation of whole blood cells responses to inflammatory stimuli in depression. Both reports are descriptive in nature, since it is currently difficult to provide a mechanistic explanation to these phenomena. We can hypothesize that factors controlling COX-2 (and perhaps IL-6) gene expression are altered in MDD, resulting in both up- and down-regulation of inflammatory mediator production. In our study, the lack of a concomitant HPA impairment would suggest that the glucocorticoid-GRE system is not involved. The diverging pattern of gene expression of COX-2 compared to inflammatory cytokines, all of which are inhibited by glucocorticoids [24], [25], would also exclude that the latter play any role. Here we also tested the hypothesis that a deregulation occurs at the level of TLR expression. However, we found no difference between TLR-4 gene expression in monocytes form healthy controls and patients, and LPS produced the same mild increase in both groups, suggesting that any difference in regulation occurring in depressed patients is to be located somewhere downstream receptor activation.

## Conclusion

In conclusion, our findings clearly indicate that MDD patients present an altered response of blood monocytes to a standardized endotoxin challenge. This ex-vivo/vitro model appears to be a promising marker of the immune dysreactivity associated to depression. It appears to be independent from the time of day, nutritional and exercise status, and HPA axis function. We also observed differences in monocyte reactivity after a standard drug treatment; however, the experimental design adopted here does not allow to draw any conclusion as to whether monocyte reactivity to endotoxin might also be used as a marker of the outcome of pharmacological treatments, since a 8-month FU in the group of healthy controls was not carried out.
